# Children’s Navigation of Contextual Cues in Peer Transgressions: The Role of Aggression Form, Transgressor Gender, and Transgressor Intention

**DOI:** 10.3389/fpsyg.2022.813317

**Published:** 2022-03-16

**Authors:** Andrea C. Yuly-Youngblood, Jessica S. Caporaso, Rachel C. Croce, Janet J. Boseovski

**Affiliations:** University of North Carolina at Greensboro, Greensboro, NC, United States

**Keywords:** physical aggression, relational aggression, moral development, intentionality, gender

## Abstract

When faced with transgressions in their peer groups, children must navigate a series of situational cues (e.g., type of transgression, transgressor gender, transgressor intentionality) to evaluate the moral status of transgressions and to inform their subsequent behavior toward the transgressors. There is little research on which cues children prioritize when presented together, how reliance on these cues may be affected by certain biases (e.g., gender norms), or how the prioritization of these cues may change with age. To explore these questions, 138 5- to 7-year-olds (younger children) and 8- to 10-year-olds (older children) evaluated a series of boy and girl characters who partook in physical or relational aggression with ambiguous or purposeful intent. Children were asked to provide sociomoral evaluations (i.e., acceptability, punishment, and intention attribution judgments) and social preferences. Transgressor gender only impacted children’s social preferences. Conversely, aggression form and transgressor intent shifted children’s sociomoral judgments: they were harsher toward physical transgressors with purposeful intent over those with ambiguous intent but made similar evaluations for relational transgressors regardless of intentionality. The present results suggest that gender is perhaps not uniformly relevant to children across all contexts, as other cues were prioritized for children’s sociomoral judgments. Since children likely have less familiarity with relational aggression compared to physical aggression, it follows that intent would only shift judgments about physical transgressors. This research provides insight about how children simultaneously navigate multiple cues in aggression contexts, which is likely reflective of their real-world experiences.

## Introduction

As children’s social worlds grow in complexity, children experience a variety of peer transgressions, such as aggressive acts, and must evaluate these transgressions as they occur. At the same time, children prefer individuals in their own social categories (e.g., gender; Maccoby, 1988; Martin and Fabes, 2001; Halim, 2016) and learn the social norms of group interaction and cohesion. Gender is a salient and fundamental social category that drives children’s social decision-making by preschool age, including their attitudes and predictions about others (e.g., Ruble et al., 2006; Arthur et al., 2008). Accordingly, preschoolers are aware of the normative behaviors and preferences of their gender ingroup (e.g., Ruble et al., 2006; Halim, 2016). In fact, some findings suggest that children attend to gender at an earlier age than they attend to other social categories, such as race, when they reason about other people (e.g., Shutts et al., 2010; Shutts, 2015; Weisman et al., 2015). Therefore, when transgressions occur, children might view these actions through the lens of gender norms as they consider a multitude of other situational cues (e.g., intentionality cues) to evaluate the moral status of an aggressive violation (e.g., Margoni and Surian, 2017; Yoo and Smetana, 2019). Beyond moral judgments, children may also use these situational cues to make decisions about whom they choose to befriend, as they may decide not to affiliate with an individual who is disruptive to group cohesion (Hitti et al., 2014). Importantly, the relevance of these cues and their interactions likely shift with age, thereby altering children’s sociomoral judgments and social preferences from early to middle childhood.

The current study examined the role of gender norms on children’s moral evaluations and social preferences in aggression contexts. Five- to 10-year-olds were presented with a series of vignettes that included three situational cues—type of aggression (i.e., aggression form), transgressor gender, and transgressor intentionality. Importantly, these cues emulate children’s knowledge, experiences, and biases regarding peer conflict and social norms (Grant and Mills, 2011). In fact, type of aggression, transgressor gender, and transgressor intentionality have been individually shown to affect children’s sociomoral reasoning (e.g., Giles and Heyman, 2005; Killen et al., 2011; Smetana and Ball, 2018). The relevance of these cues for sociomoral judgments may shift across development, as children’s experiences with different forms of aggression (e.g., Alink et al., 2006; Orpinas et al., 2015), their adherence to gender stereotypic beliefs (e.g., Conry-Murray and Turiel, 2012), and their ability to attribute intentionality (e.g., Killen et al., 2011) all change across early and middle childhood.

Few studies have examined these cues in concert, and it is unclear which cues children prioritize when asked to make sociomoral judgments. Certain contextual cues, such as lack of information about transgressor intent, may lead children to rely on other types of cues and leave space for children to use informational processing biases in their judgments (Crick and Dodge, 1996; Heyman, 2001; Boseovski et al., 2013). Other contextual cues, such as transgressor gender, may be particularly salient when the form of aggression violates children’s beliefs about gender norms and aggression (e.g., a girl who commits a physically aggressive act; Giles and Heyman, 2005; Ruble et al., 2006). Consequently, it is important to examine how children balance these various situational cues across childhood, especially given that children must manage all of these cues during real-life transgression scenarios.

### Aggression Form

To begin, it is critical to establish how children perceive different forms of aggression, regardless of gender or intentionality cues. By early childhood, children differentiate whether a transgression was characterized by sabotage to relationships (i.e., relational aggression) or overt physical harm (i.e., physical aggression; Björkqvist et al., 1992; Crick and Grotpeter, 1995). Generally, 4- to 10-year-olds judge physical aggression to be more serious, more harmful, and more deserving of punishment than relational aggression (Murray-Close et al., 2006; Smetana and Ball, 2018), perhaps because physical harm is construed as serious in most cultures and results in physical, observable distress. Physical and relational aggression also differ in the frequency that they occur in children’s everyday contexts, and this frequency changes with age. Physical aggression is more common in preschool and kindergarten than in elementary school, and acts of physical aggression peak at 3.5 years of age for the majority of children (NICHD Early Child Care Research Network, 2004; Alink et al., 2006). The use of physical aggression parallels how preschoolers’ friendships center on physical activities and proximity, along with physical descriptions that include sharing toys or holding hands (e.g., Selman et al., 1977).

Although relational aggression is also observed in the preschool classroom (e.g., Perry et al., 2021), it does not reach its peak occurrence until middle school (Orpinas et al., 2015). In addition, the types of relational aggression observed in preschool tend to be much less sophisticated (and possibly less harmful) than the types of relational aggression that occur in middle and late childhood (Ostrov et al., 2018). Also, parents and teachers tend to condemn acts of physical aggression more frequently and harshly than acts of relational aggression in the preschool years (Swit et al., 2018).

Consequently, younger children generally have more experience with physical aggression (as both victims and transgressors) than with relational aggression. This is particularly important because children actively construct moral concepts from the information they receive from their environments and daily experiences (Dahl, 2018; Smetana et al., 2018). Younger children may view acts of physical aggression as more damaging to group harmony than relational aggression, especially since they prioritize the physical dimensions of friendship (e.g., Selman et al., 1977). As children age, their social goals become more relational to enhance peer relationships and maintain group cohesion (Crick and Dodge, 1996). Older children likely have more knowledge regarding relational aggression and may be more sensitive to the harm caused by relational aggression compared to younger children. In fact, older children tend to attribute more negative intent and have more complex explanations for relational transgressions compared to their younger counterparts (Boseovski et al., 2013). An improved understanding of relational harm may lead older children to harsher evaluations of relational transgressions over physical transgressions (e.g., Yoo and Smetana, 2019), at least in comparison to younger children. Still, a variety of other situational cues are likely evident in aggression contexts, such as transgressor gender and attention to whether a transgressor behaves in line with gender norms.

### Transgressor Gender

There are a few noted gender differences in the perpetration of physical and relational aggression. Boys are more likely to engage in physical aggression than girls (e.g., Lansford et al., 2012; Perry et al., 2021). Although there is mixed evidence that girls engage in relational aggression more than boys (for a review, see Card et al., 2008), girls are more likely to engage in relational aggression compared to physical aggression (e.g., Ostrov et al., 2014). As such, children stereotypically categorize physical aggression as a characteristic of boys and relational aggression as a characteristic of girls (e.g., Giles and Heyman, 2005; Ruble et al., 2006; Martin and Ruble, 2010). This follows children’s more general stereotypes that regard boys as fighters/hitters who are rough and physically active, but girls as gentle/passive, with relationships that center on intimate, personal experiences (e.g., Maccoby, 1990, 2004; Basu, 1991; Ruble et al., 2006; Miller et al., 2009). Past literature establishes that these gender norms permeate children’s judgments about others, including individuals in aggression contexts (e.g., Crick et al., 1996; Giles and Heyman, 2005). As further support for this idea, children are more likely to misremember “mismatched” aggressive situations (i.e., a boy being relationally aggressive or a girl being physically aggressive) than those that are matched (i.e., a boy being physically aggressive or a girl being relationally aggressive; Giles and Heyman, 2005). Given that these characteristics reflect the group norms that children hold for their gender ingroup, they may view individuals less favorably if they behave in ways that go against group norms (Hitti et al., 2014; Mulvey et al., 2014).

Although even preschoolers report that gender norm adherence is a personal choice (Conry-Murray and Turiel, 2012; Conry-Murray et al., 2020), they also report fewer positive judgments toward individuals who behave counter-stereotypically and therefore against social group norms, at least compared to those who behave stereotypically (e.g., Blakemore, 2003). By middle to late childhood, children exhibit increasingly flexible gender attitudes and become more accepting of counter-stereotypic information (Martin, 1989; Ruble et al., 2006; Conry-Murray and Turiel, 2012). One explanation for this flexibility with development is gender essentialism, which is endorsed in early childhood but subsides by about 9 years of age (Taylor et al., 2009). Young children who endorse gender essentialism view gender as fixed and immutable, which allows them to make a variety of predictions about others based merely on gender category information (e.g., Taylor et al., 2009; Meyer and Gelman, 2016) and thus adhere to gender norms. Alternatively, since even young children understand that adhering to gender norms is a personal choice, perhaps this understanding strengthens with development and becomes more uniformly applied across contexts and situations, leading to flexible gender attitudes in middle to late childhood.

In addition, younger children view harm committed by a member of their gender ingroup as worse than harm committed by a member of their gender outgroup, whereas older children only focus on the transgression in their moral judgments (Mulvey, 2016). Nevertheless, this does not suggest that younger children prioritize gender over the moral harm implicated by a transgression. Given younger children’s heightened attention to gender as detailed above, one interpretation is that the harm committed by a gender ingroup member was perceived as betrayal and therefore more problematic than the harm committed by a gender outgroup member. Thus, it is possible that 5- to 7-year-olds will use transgressor gender cues more than 8- to 10-year-olds, particularly in situations when the transgressor commits a form of aggression that counters norms for their gender group (e.g., a girl who is physically aggressive; Giles and Heyman, 2005). Still, reliance on gender cues will likely change if the transgressor’s intent is unclear vs. purposeful.

### Transgressor Intentionality

The use of both aggression form and transgressor gender cues may depend on the extent to which the transgression was committed with clear intent. Intentionality cues affect how children process social situations and their subsequent behaviors toward others, and the influence of intention extends to contexts beyond aggression. For example, children as young as 3 years of age selectively choose to help individuals with helpful versus harmful intentions (Vaish et al., 2010). Further, and compared to 3-year-olds, 5-year-olds choose to distribute fewer resources to an actor with negative intentions and judge how right or wrong a behavior is based on an actor’s intentions (Li and Tomasello, 2018). Preschoolers also distribute fewer resources to actors who take resources rather than give away resources (Vogelsang and Tomasello, 2016). In aggression contexts, intention influences children’s moral judgements of transgressions (Killen et al., 2011), transgressor trait and emotion attributions (Boseovski et al., 2013), and how children ultimately respond to transgressions (Lansford et al., 2006). By 5 years of age, children are readily able to incorporate intentionality information into their sociomoral judgments (e.g., Zelazo et al., 1996; Killen et al., 2011; Cushman et al., 2013). The ability to weigh intentionality in conjunction with other relevant cues (e.g., foreseeability and trait information) continues to develop through middle childhood (Yuill and Perner, 1988; Heyman and Gelman, 1998; Killen et al., 2011).

The degree to which a behavior is clearly intentional or unintentional impacts children’s related judgments (e.g., Zelazo et al., 1996; Heyman and Gelman, 1998; Grant and Mills, 2011; Cushman et al., 2013). Children attribute more negative causal and trait attributions when a transgression is purposeful compared to when intent is ambiguous (Boseovski et al., 2013). In turn, children also judge intentional actions as more morally wrong and believe that they cause more harm than accidental transgressions (Killen et al., 2011). Consequently, no matter what the form of aggression or the gender of the transgressor, children may unilaterally condemn intentional transgressions. However, there is less consensus on how children judge acts in which intentionality is ambiguous. Children may rely on other situational cues, such as aggression form or transgressor gender, more heavily in these scenarios. Indeed, children tend to rely on gender cues when presented with characters whose intentions are ambiguous (i.e., unclear if the person behaved purposely): children evaluate boys’ ambiguous behaviors more negatively than the same ambiguous behavior by girls (Heyman, 2001; Giles and Heyman, 2004). This could result from expectations that boys often engage in rough behavior that could lead to physical aggression. Thus, the current study sought to clarify the role of intent, among other cues, on children’s judgments about transgressors.

### The Current Study

In the current study, the impact of different contextual cues (aggression form, transgressor gender, and transgressor intentionality) was investigated to gain a better understanding of the relative importance of each cue on children’s sociomoral judgments, including age-related changes. We were particularly interested in whether gender norms significantly impacted children’s sociomoral evaluations of transgressors and how the relevance of gender shifted for different forms of aggression or as a function of transgressor intentionality. To accomplish this, 5- to 10-year-olds were presented with four transgression stories: a boy perpetrating relational aggression, a girl perpetrating relational aggression, a boy perpetrating physical aggression, and a girl perpetrating physical aggression. All stories depicted intention as either unambiguous (i.e., stories mentioned that the transgressor behaved aggressively on purpose) or ambiguous (i.e., stories mentioned that the transgressor behaved aggressively with no mention of intentionality). Children’s reasoning was assessed through acceptability, deserved punishment, and intention attribution ratings. In addition, children were asked a social preference question to gauge how much they would like to befriend each transgressor. The addition of the social preference question provided information about how children view the person who committed the transgression, rather than focusing on the transgression itself. This may have implications for who children would include or exclude from their social groups: a preference for one transgressor over another could suggest which transgressor children would rather include in their group despite aggressive behavior, along with whose exclusion children might regard as more or less acceptable.

Because the goal of the current study was to examine how these cues interact, our primary hypotheses focused on interactions between aggression form, transgressor gender, transgressor intentionality, and age. To begin, we expected an interaction between aggression form, transgressor gender, and transgressor intentionality. We predicted that children would be more attentive to transgressor gender and aggression form cues in the ambiguous condition due to the absence of explicit information about intentionality (e.g., Crick and Dodge, 1996; Heyman, 2001; Giles and Heyman, 2004). That is, we expected that children across both age groups in the unambiguous condition would rate the acts more harshly across all judgment questions without differentiating their judgments based on aggression form and transgressor gender, whereas children in the ambiguous condition would rate the transgressions differently based on aggression form and transgressor gender.

Next, we expected an interaction between age and aggression form: older children were predicted to report harsher judgments toward relational transgressors than younger children. Although both age groups were expected to view physical harm as serious, only older children were expected to perceive relational harm as equally wrong to physical harm because of the damage it could inflict on social group cohesion. This was predicted due to increasingly complex explanations for relational transgressions with age (e.g., Boseovski et al., 2013), along with the increase in experience with relational aggression compared to the decrease in experience with physical aggression (e.g., Alink et al., 2006; Orpinas et al., 2015).

Finally, we predicted an interaction between age, aggression form, and transgressor gender. Because children are generally less favorable toward gender counter-stereotypical behavior (e.g., Blakemore, 2003), we expected that children would make harsher judgments about transgressors who behaved counter to gender norms (i.e., a physically aggressive girl and a relationally aggressive boy). We further expected that children would report less willingness to befriend a transgressor who behaved in a counter-stereotypical way because the counter-stereotypical act would violate group norms (e.g., Hitti et al., 2014). However, these patterns were expected to dampen with age due to increasingly flexible gender attitudes in middle to late childhood (see Ruble et al., 2006 for review).

## Materials and Methods

### Participants

In total, 138 5- to 10-year-olds were tested: 68 younger children (5- to 7-year-olds; 36 girls and 32 boys; *M* = 6.01, *SD* = 0.84) and 70 older children (8- to 10-year-olds; 35 girls and 35 boys; *M* = 9.01, *SD* = 0.81). Participants were recruited from a developmental laboratory database in a mid-sized city, and the majority were from middle- to upper-class families. Participants’ racial backgrounds were reported as follows: 58% White, 15.2% Black, 1.4% Asian, 15.2% identified as mixed race, 2.2% identified as other, and 8% chose not to report their racial background.

### Materials

Children were shown cartoon pictures of boys and girls participating in transgressions on a computer screen. Each scenario had three sets of pictures that outlined what occurred with both photos and words. Children were shown photos of children playing board games or cards in a classroom for relational aggression and photos of children playing catch with a basketball or baseball outdoors for physical aggression. In all scenarios, victims displayed a sad affect after the transgression and friends of the transgressor displayed no affect. Transgressors had an angry face when intent was purposeful (unambiguous condition) and transgressors displayed neutral affect when intent was ambiguous (ambiguous condition).

### Design

**FIGURE 1:**
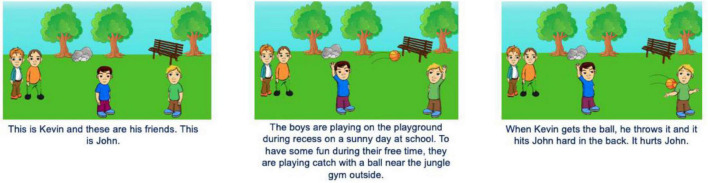
Example story depicting physical aggression in the ambiguous condition. This story is depicted with boy characters.

**FIGURE 2:**
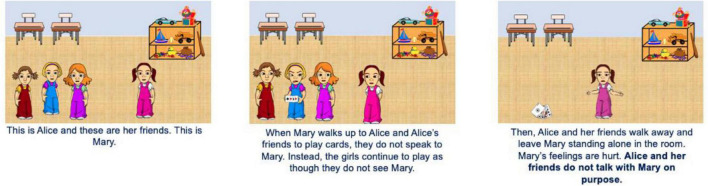
Example story depicting relational aggression in the unambiguous condition. This story is depicted with girl characters.

A mixed design was used with age (5- to 7-year-olds vs. 8- to 10-year-olds) and ambiguity condition (unambiguous: purposeful intent vs. ambiguous: ambiguous intent) as between-subject variables. Aggression form (relational vs. physical) and transgressor gender (girl vs. boy) were within-subject variables. Children in the unambiguous condition were explicitly told that the transgressor acted on purpose, but intent information was left out of the ambiguous condition. All children were shown two instances of relational aggression (one with a boy transgressor, one with a girl transgressor) and two instances of physical aggression (one with a boy transgressor, one with a girl transgressor). Thus, children saw boys and girls who engaged in aggression that aligned with (girls: relational aggression, boys: physical aggression) or countered (girls: physical aggression, boys: relational aggression) gender norms. For each instance of relational aggression, the transgressor and their friends ignored a peer’s request to play. For each instance of physical aggression, the transgressor hit someone with a ball. The victims in all stories matched the transgressor’s gender (refer to Figures 1, 2).

### Procedure

Children with signed parental consent forms were tested in private rooms in their school or a developmental laboratory. Researchers obtained verbal assent from all participants and written assent from participants of 7 years of age and older. Testing took approximately 20 min.

Prior to testing, a researcher introduced herself and told the child she would be telling stories to which there were no right or wrong answers. Following their assent, children were presented with four stories. Ambiguity condition was counterbalanced. The presentation order for aggression form (i.e., physical, relational) was counterbalanced.

For relational aggression, children were presented with two stories. One story included a group of boys and a boy transgressor and the other included a group of girls and a girl transgressor (adapted from Boseovski et al., 2013). Children were shown photos of the transgressor and two friends playing a game as a victim stood nearby. Game type (board game or card game) was randomized. Importantly, children were told, “When [victim] walks up to [transgressor] and [transgressor]’s friends, they do not speak to [victim]. Instead, the boys/girls continue to play as though they do not see [victim].” Children then saw a photo of the victim, sad and alone in the room, and were told, “Then, [transgressor] and his/her friends walk away and leave [victim] standing alone in the room. [Victim]’s feelings are hurt.”

For physical aggression, children were presented with two stories. One story included a group of boys and a boy transgressor and the other included a group of girls and a girl transgressor (adapted from Dodge, 1980). Children were shown photos of the transgressor and victim playing catch. Type of activity (basketball or baseball) was randomized. Importantly, children were told, “When [transgressor] gets the ball, he/she throws it and it hits [victim] hard in the back. It hurts [victim].”

In the unambiguous condition, each relational or physical aggression story concluded with the researcher stating the transgressor’s actions were committed on purpose. Transgressor intent information was not included in the ambiguous condition. Further, transgressors displayed negative as opposed to neutral affect to emphasize their intent in the unambiguous condition.

#### Dependent Measures

After each story, children were asked the following questions.

##### Acceptability

Children evaluated the acceptability of each transgression.^1^ For relational aggression, children were asked, “How bad was it for [transgressor] to continue to play as though he/she didn’t see [victim], and then leave [victim] alone in the room?” For physical aggression, children were asked “How bad was it for [transgressor] to throw the ball and hit victim hard in the back?” Children used a 5-point visual Likert scale to respond. Answers were scored as follows: 1 = not at all, 2 = a little, 3 = sort of, 4 = a lot, and 5 = a whole lot.

##### Punishment

Children were asked whether the transgressor should get in trouble. For relational aggression, children were asked, “Should [transgressor] get in trouble for continuing to play as though he/she didn’t see [victim] and leaving [victim] alone in the room?” For physical aggression, children were asked “Should [transgressor] get in trouble for throwing the ball and hitting [victim] hard in the back?” Children responded no (scored as 0) or yes (scored as 1).

##### Intention Attributions

Children were asked how purposefully each transgressor acted, which represented how much intent children attributed to the transgressor, above and beyond the intent manipulation (i.e., inclusion of whether the transgressor behaved on purpose). For relational aggression, children were asked, “How much did [transgressor] try to continue to play as though he/she didn’t see [victim], and then leave [victim] alone in the room?” For physical aggression, children were asked “How much did [transgressor] try to throw the ball and hit [victim] hard in the back?” Children used a 5-point visual Likert scale to respond. Answers were scored as follows: 1 = not at all, 2 = a little bit, 3 = sort of, 4 = a lot, and 5 = a whole lot.

##### Social Preferences

Children were asked how much they wanted to befriend each transgressor (i.e., “How much would you want to be friends with [transgressor]?”). Children used a 5-point visual Likert scale to respond. Answers were scored as follows: 1 = not at all, 2 = a little, 3 = sort of, 4 = a lot, and 5 = a whole lot.

## Results

A single 2 (age: 5–7.9 or 8–10 years) × 2 (ambiguity condition: unambiguous or ambiguous) × 2 (aggression form: relational vs. physical) × 2 (transgressor gender: girl vs. boy) × 2 (participant gender: girls vs. boys) mixed ANOVA was conducted for each continuous measure (i.e., acceptability, intention attributions, and social preferences). Generalized estimating equations (GEE) were used to conduct binary repeated measures logistic regression for dichotomous measures (i.e., punishment). For follow-up tests, Holm–Bonferroni corrections were used to minimize the risk of type I error.

A Monte Carlo simulation for factorial experimental designs and follow-up pairwise comparisons (refer to Lakens and Caldwell, 2021) revealed sufficient power for a two-way interaction (90% power), but insufficient power for a three-way interaction (less than 80% power). Thus, any null findings for three-way interactions should be interpreted with caution.

### Acceptability

**FIGURE 3:**
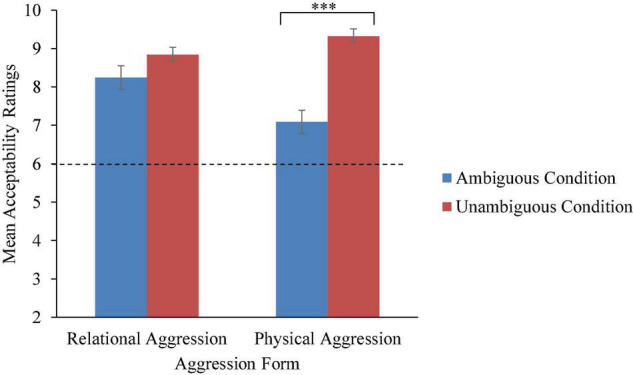
Mean acceptability ratings by aggression form and ambiguity condition across age groups. Error bars represent SEs. Note that acceptability ratings were coded as follows: 1, not at all; 2, a little; 3, sort of; 4, a lot; and 5, a whole lot. Ratings were summed across transgressor gender, resulting in a range of 2–10. *** Indicates a significant difference (*p* < 0.001) between the ambiguous and unambiguous conditions.

A significant interaction between transgressor gender, aggression form, and ambiguity condition was anticipated, but not supported. Although not expected, there was a significant aggression form × ambiguity interaction, *F*(1, 133) = 17.71, η*_*p*_*^2^ = 0.12, *p* < 0.001 (refer to Figure 3). To interpret the interaction, follow-up tests with Holm–Bonferroni corrections revealed that relational transgressions were evaluated as similarly bad in the unambiguous (*M* = 8.84, *SD* = 1.58) and ambiguous conditions (*M* = 8.26, *SD* = 2.53), *t*(136) = 1.61, *p* = 0.11. Children were more likely than expected by chance to report that the relational transgressions were very bad regardless of ambiguity condition, *ps* < 0.001. Conversely, children reported that physical transgressions in the unambiguous condition (*M* = 9.32, *SD* = 1.33) were significantly worse than physical transgressions in the ambiguous condition (*M* = 7.09, *SD* = 2.65), *t*(102) = 6.25, *p* < 0.001. Still, children were more likely than expected by chance to report that the physical transgressions were very bad regardless of ambiguity condition, *ps* ≤ 0.001.

A significant interaction between aggression form and age group was hypothesized, but not found. Although not anticipated, there was a significant main effect of age group, *F*(1, 133) = 7.19, η*_*p*_*^2^ = 0.05, *p* = 0.008. Follow-up tests suggested that older children (*M* = 17.60, *SD* = 2.79) evaluated transgressions as significantly worse than younger children (*M* = 15.84, *SD* = 4.36), *t*(113) = − 2.82, *p* = 0.01. Overall, both age groups were more likely than expected by chance to rate the transgressions as bad, *ps* < 0.001.

A significant interaction between transgressor gender, aggression form, and age group was expected, but did not emerge, *p* > 0.05.

### Punishment

**FIGURE 4:**
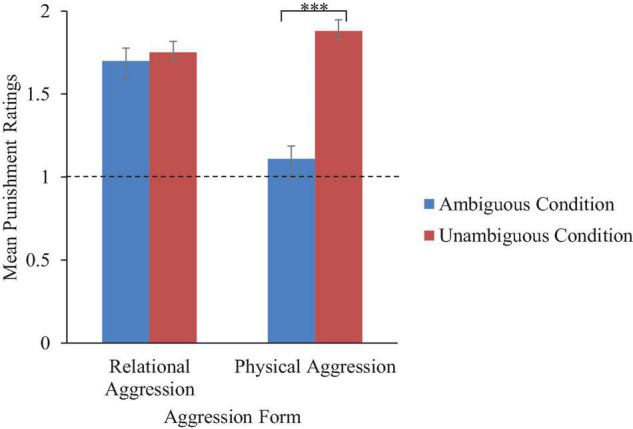
Mean punishment ratings by aggression form and ambiguity condition across age groups. Error bars represent SEs. Note that acceptability ratings were coded as follows: 0, no; 1, yes. Ratings were summed across transgressor gender, resulting in a range of 0–2. *** Indicates a significant difference (*p* < 0.001) between the ambiguous and unambiguous conditions.

A significant interaction between transgressor gender, aggression form, and ambiguity condition was hypothesized, but not found. Although not hypothesized, there was a significant aggression form × ambiguity interaction, Wald χ^2^ = 16.63, OR = 10.77, *p* < 0.001. To interpret the interaction, follow-up tests with Holm-Bonferroni corrections indicated that punishment ratings were similar for relational transgressors in the unambiguous (*M* = 1.75, *SD* = 0.56) and ambiguous (*M* = 1.70, SD = 0.65) conditions, *t*(136) = 0.49, *p* = 0.63. Children were more likely than expected by chance to report that relational transgressors in the unambiguous and ambiguous conditions should be punished, *ps* < 0.001. However, punishment ratings were higher for physical transgressors in the unambiguous condition (*M* = 1.88, *SD* = 0.41) compared to the ambiguous condition (*M* = 1.11, *SD* = 0.93), *t*(95) = 6.34, *p* < 0.001. Children in the unambiguous condition were more likely than expected by chance to report that physical transgressors should be punished, *t*(67) = 17.91, *p* < 0.001, yet children in the ambiguous condition did not systematically report that physical transgressors should be punished, *t*(69) = 1.03, *p* = 0.31 (refer to Figure 4).

A significant interaction between aggression form and age group was predicted, but not supported, *p* > 0.05.

A significant interaction between transgressor gender, aggression form, and age group was anticipated, but did not emerge, *p* > 0.05.

### Intention Attributions

A significant interaction between transgressor gender, aggression form, and ambiguity condition was hypothesized, but not found, *p* > 0.05.

**FIGURE 5:**
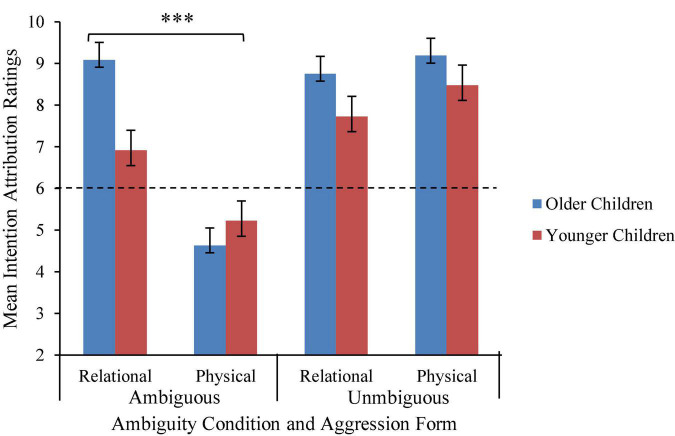
Mean intention attribution ratings by aggression form, ambiguity condition, and age group. Error bars represent SEs. Note that intention attributions were coded as follows: 1, not at all; 2, a little; 3, sort of; 4, a lot; and 5, a whole lot. Ratings were summed across transgressor gender, resulting in a range of 2–10. *** Indicates a significant age × aggression form interaction (*p* < 0.001).

A significant interaction between aggression form and age group was expected, but not supported. Unexpectedly, there was a significant interaction between age group, aggression form, and ambiguity condition, *F*(1, 128) = 6.56, η*_*p*_*^2^ = 0.05, *p* = 0.01 (refer to Figure 5 and Table 1). To interpret the interaction, follow-up tests were conducted with Holm–Bonferroni corrections. In the unambiguous condition, there was no significant interaction between aggression form and age group, *F*(1, 65) = 0.20, η*_*p*_*^2^ = 0.00, *p* = 0.65. In the unambiguous condition, children in each age group reported that the relational and physical transgressors acted purposefully, *ps* < 0.001. However, in the ambiguous condition, there was a significant aggression form by age group interaction, *F*(1, 67) = 12.62, η*_*p*_*^2^ = 0.16, *p* < 0.001. Compared to younger children (*M* = 6.92, *SD* = 2.90), older children (*M* = 9.09, *SD* = 1.36) reported that relational transgressors acted more purposefully, *t*(52) = − 4.08, *p* < 0.001. Younger children’s reports did not differ significantly from chance, *t*(35) = 1.93, *p* = 0.06, while older children’s ratings were above chance, *t*(32) = 13.11, *p* < 0.001. By contrast, intention attributions for physical transgressors did not differ between younger children (*M* = 5.22, *SD* = 2.09) and older children (*M* = 4.64, *SD* = 2.38), *t*(67) = 0.93, *p* = 0.36. Older children were less likely than expected by chance to report that the physical transgressors behaved purposefully, *t*(32) = − 3.29, *p* = 0.002. Younger children did not respond systematically, *t*(35) = − 1.66, *p* = 0.11.

**TABLE 1:** Means and SDs for intention attribution question [“How much did (transgressor) try…?”].

	Unambiguous				Ambiguous			
	Relational		Physical		Relational		Physical	
Age group	*n*	*M* (*SD*)	*n*	*M* (*SD*)	*N*	*M* (*SD*)	*n*	*M* (*SD*)
Younger	30	7.73 (2.61)	31	8.48 (2.06)	37	6.92 (2.90)	36	5.22 (2.81)
Older	37	8.76 (1.94)	37	9.19 (1.10)	33	9.09 (1.35)	33	4.64 (2.38)

*Intention attributions were summed across transgressor gender. Range: 2–10.*

A significant interaction between transgressor gender, aggression form, and age group, was predicted, but did not emerge, *p* > 0.05.

### Social Preferences

A significant interaction between transgressor gender, aggression form, and ambiguity condition was hypothesized, but not supported. Although not anticipated, there was a significant aggression form × ambiguity interaction, *F*(1, 133) = 28.03, η*_*p*_*^2^ = 0.17, *p* < 0.001. To interpret the interaction, follow-up tests were conducted with Holm–Bonferroni corrections. Children reported a higher desire to befriend relational transgressors in the ambiguous condition (*M* = 3.86, *SD* = 2.29) compared to the unambiguous condition (*M* = 3.07, *SD* = 1.57), *t*(122) = − 2.35, *p* = 0.02. Children in the unambiguous and ambiguous conditions were less likely than expected by chance to report desire to befriend the relational transgressors overall, *ps* < 0.001. Further, children reported a higher desire to befriend physical transgressors in the ambiguous condition (*M* = 5.51, *SD* = 2.64) compared to the unambiguous condition (*M* = 2.93, *SD* = 1.36), *t*(104) = − 7.27, *p* < 0.001. Children were less likely than expected by chance to report desire to befriend physical transgressors in the unambiguous condition, *t(*67) = − 18.58, *p* < 0.001. However, children’s ratings did not differ significantly from chance in the ambiguous condition, suggesting a relatively neutral desire to befriend physical transgressors, *t*(69) = − 1.54, *p* = 0.128.

A significant interaction between aggression form and age group was predicted, but not found. However, and unexpectedly, there was a main effect of age group, *F*(134) = 5.64, η*_*p*_*^2^ = 0.04, *p* = 0.02. Collapsed across aggression form, transgressor gender, and transgressor intentionality, younger children (*M* = 8.54, *SD* = 4.55) expressed a greater desire to befriend the transgressors than older children (*M* = 6.90, *SD* = 2.90), *t*(113) = 2.52, *p* = 0.01. Still, children from both age groups were less likely than expected by chance to express a strong desire to befriend the transgressors overall, *ps* < 0.01.

A significant interaction between transgressor gender, aggression form, and age group was anticipated, but did not emerge. Unexpectedly, there was a significant aggression form × transgressor gender interaction, *F*(1, 133) = 10.20, η*_*p*_*^2^ = 0.07, *p* = 0.002. To interpret the interaction, follow-up tests were run with Holm–Bonferroni corrections. Children reported a higher desire to befriend the relational boy transgressor (*M* = 1.88, *SD* = 1.23), compared to the relational girl transgressor (*M* = 1.59, *SD* = 1.06), *t*(137) = 2.93, *p* = 0.004. However, children’s desire for friendship did not differ between the physical boy transgressor (*M* = 2.04, *SD* = 1.35) and physical girl transgressor (*M* = 2.20, *SD* = 1.33), *t*(137) = − 1.70, *p* = 0.09 did not differ. Children were less likely than expected by chance to report desire to befriend the relational boy or girl transgressors or the physical boy or girl transgressors, *ps* < 0.01.

#### Additional Findings

The mixed ANOVA for the social preferences measure revealed a participant gender × transgressor gender interaction, *F*(133) = 9.92, η*_*p*_*^2^ = 0.07, *p* = 0.002. We did not anticipate this interaction, so these results are exploratory and should be interpreted with caution. Follow-up tests with Holm–Bonferroni corrections revealed that boys (*M* = 4.37, *SD* = 2.16) reported a higher desire to befriend the boy transgressors than girls (*M* = 3.49, *SD* = 2.06), *t*(134) = 2.45, *p* = 0.02. Conversely, girls (*M* = 3.73, *SD* = 2.10) and boys (*M* = 3.85, *SD* = 1.88) did not differ in their desire to befriend the girl transgressors, *t*(136) = 0.35, *p* = 0.73. Both boys and girls were less likely than expected by chance to report desire to befriend either boy or girl transgressors, *ps* < 0.001.

## Discussion

The present study examined which cues (aggression form, transgressor gender, and transgressor intentionality) children prioritized to guide their sociomoral reasoning about transgressors. We explored whether ambiguous transgressor intent led children to prioritize aggression form and transgressor gender cues to guide their sociomoral judgments, such as whether behaviors that were misaligned with gender norms and thus violated group cohesion would be judged more harshly than behaviors that aligned with gender norms. We also explored how reliance on these cues differed between 5- to 7-year-olds and 8- to 10-year-olds, as the prevalence of different aggression forms changes across childhood (Alink et al., 2006; Orpinas et al., 2015), along with children’s endorsement of gender norms (e.g., Ruble et al., 2006) and perceptions of intentionality (e.g., Killen et al., 2011). Although past literature considered each of these cues (e.g., Crick et al., 1996; Giles and Heyman, 2005; Boseovski et al., 2013), this study was the first to experimentally investigate all three cues concurrently.

Despite our initial hypotheses, aggression form, transgressor gender, and transgressor intentionality did not interact to guide children’s sociomoral judgments. Only aggression form and transgressor intentionality guided children’s sociomoral decision making (i.e., acceptability, punishment, and intention attribution ratings): children across age groups evaluated physical aggression more harshly when intent was purposeful, but intent did not influence children’s relational aggression evaluations. Although transgressor gender did not substantially influence children’s sociomoral judgments, it was relevant to children’s social preferences: across age groups, children reported a greater desire to befriend the relational boy transgressor than the relational girl transgressor. However, the present study did not measure children’s perceptions of gender norms in aggression contexts. Therefore, it is unclear whether the effect of transgressor gender was due to transgressor gender or transgressor gender in conjunction with whether the transgressor partook in gender normative behavior. Regardless, the present results suggest that transgressor gender (whether on its own or together with gender normative behavior) was more relevant to children’s *attitudes* about the transgressors (i.e., social preferences) than their sociomoral judgments about the transgressor’s *actions*. Lastly, although interactions with age group were limited to intention attributions, sociomoral judgments were harsher among 8- to 10-year-olds than 5- to 7-year-olds, and older children reported less desire to befriend the transgressors. This likely persisted due to older children’s better ability to successfully integrate the multitude of cues presented.

### Sociomoral Judgments: Which Cues Matter?

Previous research suggests that intent ambiguity could lead children to focus on other contextual cues (e.g., Boseovski et al., 2013), and the present study supplements this idea by illustrating that the absence of intent information does not necessitate the use of *all* other cues provided. We hypothesized that all three cues provided (aggression form, transgressor gender, and transgressor intentionality) in the present study would interact to influence children’s sociomoral judgments, but children only relied on aggression form and transgressor intentionality. Critically, this pattern mostly persisted across age groups.

The minimized role of transgressor gender is surprising because gender is a salient and relevant social category that drives children’s social decision-making (e.g., Halim and Ruble, 2010), along with the fact that children are attentive to information that facilitates group cohesion (e.g., Hitti et al., 2014; Mulvey et al., 2014). Specifically, physical aggression is more often associated with boys and relational aggression is more often associated with girls (e.g., Giles and Heyman, 2005). However, past findings also reflect that young children view gender norm adherence as a personal choice, whereas partaking in aggression is morally wrong (e.g., Conry-Murray and Turiel, 2012; Smetana et al., 2014). Thus, the general harm implicated by physical and relational aggression potentially led children in the present study to disregard transgressor gender and instead focus on the action committed for their sociomoral judgments. It follows that aggression form and transgressor intentionality would be relevant to children’s sociomoral evaluations across age groups. It is possible that the role of transgressor gender, or transgressor gender together with gender normative behavior, would be better captured with a measure related to children’s gender normative beliefs in aggression contexts. Still, the role of aggression form and transgressor intentionality holds, regardless of this limitation.

Overall, and consistent with previous literature (e.g., Murray-Close et al., 2006), children across age groups generally made harsher ratings toward physical transgressors with purposeful intent (unambiguous condition) compared to those with ambiguous intent (ambiguous condition). Although this was not explicitly hypothesized, intention cues were likely prioritized for physical aggression due to extensive experience and understanding of physical aggression from a young age, at least compared to relational aggression. Not only is physical aggression more readily observable (i.e., someone is visibly hit or hurt), but physical aggression occurs at higher rates of frequency during early childhood (e.g., Alink et al., 2006), and children are more likely to receive moral messages about physical aggression from parents and teachers compared to relational aggression (Swit et al., 2018). Preschoolers also rate physically aggressive behaviors as wrong regardless of rules, authority, or cultural context (e.g., Ball et al., 2017; Smetana and Ball, 2018). These findings are coupled with the fact that there are pervasive environmental messages that stress the harm associated with physical aggression. It follows that children’s sociomoral judgments across age groups were impacted by intentionality and therefore harsher for instances of physical aggression with purposeful intent, rather than ambiguous intent.

Further, children seek to maintain positive perceptions of others (e.g., Boseovski, 2010), which likely compounded with their extensive knowledge about physical aggression to elicit less harsh sociomoral evaluations toward physical transgressors with ambiguous intentions compared to physical transgressors with purposeful intentions. Indeed, research with adults suggests that people are motivated to base their decision-making on their expectations and desires, often in line with their biases (e.g., Kunda, 1990). Since children know that physical aggression can cause serious harm and are likely aware that physical harm is less common by the time that they reach elementary school (NICHD Early Child Care Research Network, 2004; Alink et al., 2006; Ball et al., 2017), they may be less willing to believe that physical harm is a purposeful act in the absence of explicit intent information. Instead, their general preference for positive information and perceptions may lead to assumptions that the transgression was accidental in the ambiguous condition, and they therefore discounted the seriousness of the physical transgression.

In fact, results from one type of sociomoral evaluation in the present study (intention attributions) suggest that children rated physical aggression as less intentional in the ambiguous condition, but relational aggression as intentional in the ambiguous condition. This was stronger among 8- to 10-year-olds than 5- to 7-year-olds. Importantly, this pattern was not anticipated and should be interpreted with caution. It is possible that this age difference arose due to the increased occurrence and experience with relational aggression as children progress through middle childhood (Orpinas et al., 2015). It may also reflect children’s increased abilities to integrate multiple pieces of information to make complex judgments with age. Children make use of intentionality information for their sociomoral judgments by 5 years of age (e.g., Zelazo et al., 1996) and judge intentional behavior as wrong (Killen et al., 2011). This was evident in the present study by the lack of age differences in intention attribution ratings when intent was purposeful (i.e., unambiguous). Conversely, it seems that older children were better able to jointly consider *ambiguous* intent and aggression form with age. Past literature supports this idea by demonstrating that children in middle childhood are increasingly able to consider intention information with other contextual cues (e.g., Heyman and Gelman, 1998).

To further explain the above age differences, it is critical to note that the intention attribution question required children to think about how much each transgressor tried to commit their behavior, in contrast to the other sociomoral evaluations in the present study. Acceptability judgments required children to rate how bad the transgressor’s actions were, while punishment judgments required children to decide whether the transgressor should get in trouble. Thus, children only needed to think about their *own* sociomoral beliefs. In turn, acceptability and punishment were perhaps easier for children to comprehend across age, leading to a lack of age-related interactions. Conversely, intention attribution ratings were more complex because children had to simultaneously navigate their *own* beliefs about the transgressor’s actions *and* the cues presented in the story (e.g., did the story state whether the transgressor behaved on purpose or on accident?), which was likely difficult to do when intent was ambiguous. Qualitative data (i.e., asking participants to provide a reason for their intention attributions) could verify how the cues provided in each story drove older and younger children’s intention attribution ratings.

It is important to note that children’s other sociomoral evaluations (acceptability and punishment) were similar for relational transgressors with purposeful intentions and ambiguous intentions across age groups, but this could be due to the plausibility of the act in question. Since relational aggression involves sabotage to personal relationships, rather than the overt physical harm implicated with physical aggression (e.g., Crick and Grotpeter, 1995), it is probable that relational acts in the ambiguous condition were perceived as purposeful. Indeed, the intention attribution findings above further support this idea, as relational aggression was interpreted as intentional in the ambiguous condition, yet this did not occur for physical aggression. Despite these findings and the general reliance on aggression form and transgressor intent cues for children’s sociomoral evaluations, children’s social preferences reflected a reliance on transgressor gender, suggesting a potential disconnect between social preferences and sociomoral judgments.

### Social Preferences: Which Cues Matter?

Across age groups, children expressed a greater desire to befriend relationally aggressive boys over relationally aggressive girls, but these differences did not arise for the physical transgressors. This was unexpected, given that past findings report more positive social judgments toward stereotypic over counter-stereotypic individuals (e.g., Blakemore, 2003; Halim, 2016), perhaps because stereotypic behavior facilitates group cohesion. It is unclear how much gender norms guided children’s preference for relationally aggressive boys over girls. If a lack of adherence to gender norms drove the preference for relationally aggressive boys over girls, one would expect a preference for girl transgressors over boy transgressors in physical aggression contexts. Alternatively, perhaps a preference for girls in physical aggression contexts was not found because children prioritized physical harm cues over gender norms.

Further, across age groups, children’s social preferences varied by participant gender: boys reported a greater desire than girls to befriend boy transgressors, but both boys and girls reported a low desire to befriend girl transgressors, implying that only boys were more forgiving of a fellow ingroup member committing aggression. This pattern was not hypothesized but likely emerged because boys often show stronger ingroup biases than girls (e.g., Benozio and Diesendruck, 2015). Further, past research suggests that girls make harsher judgments than boys in aggression contexts (e.g., Killen and Stangor, 2001; Goldstein et al., 2002; Murray-Close et al., 2006). Still, gender was not the only relevant cue that drove children’s social preferences.

Moreover, and in line with sociomoral judgments in the present study and in past work about purposeful intent (Killen et al., 2011; Boseovski et al., 2013), children were okay with befriending physical transgressors with ambiguous intentions, but they reported a low desire to befriend physical transgressors with purposeful intentions. It follows that children would express a higher desire to befriend physical transgressors with ambiguous intentions over purposeful intentions, as they prioritized intent and aggression form cues for other measures in the present study (as previously mentioned, they interpreted the actions by the physical transgressor with ambiguous intentions as less bad and less punishable, and they provided less harsh intention attributions). Further, because of their familiarity and experience with physical aggression, participants have likely committed accidental physical aggression at least once before or were once victims of accidental physical aggression, which may have facilitated their decisions to befriend the physical transgressor with ambiguous intentions. Although children also reported a higher desire to befriend relational transgressors with ambiguous over purposeful intentions, children did not report a strong desire to befriend either transgressor. Thus, children were forgiving of physical aggression and not relational aggression, but this only occurred in the absence of explicit intent information (i.e., ambiguous condition).

### Limitations and Future Directions

First, the achieved sample size was not enough to detect three-way interactions. It is possible that the hypothesized three-way interactions (e.g., aggression form × transgressor gender × transgressor intentionality) would be detected with a larger sample size, especially if the three-way interactions have small effects. As mentioned earlier, results regarding the three-way interactions should be taken with caution. Despite this limitation, other significant effects and interactions were found in the present study.

Further, participants’ judgments of and adherence to gender norms were not measured, which limits interpretations centered on gender normative behavior. Although gender stereotype endorsement diminishes with age (e.g., Halim and Ruble, 2010), and there were few age-based interactions in the present study, the role of gender normative behavior might be better reflected with a measure that captures how much children associate relational and physical aggression with each gender or how much children adhere to gender norms. Perhaps some children were unaware that a gender norm was violated due to low endorsement of gender norms, although this is unlikely given children’s abundant knowledge about and experience with gender and aggression. It is also possible that children who endorse gender norms the most strongly were the harshest against transgressors who behaved in contrast to gender norms and potentially violated group cohesion (i.e., relationally aggressive boys and physically aggressive girls).

Additionally, the present study matched transgressor and victim gender but the influence of transgressor gender is perhaps more evident when transgressor and victim gender are mismatched. Nevertheless, this could also introduce ingroup gender biases (e.g., Rutland et al., 2010): children might be harsher toward transgressors of their gender outgroup, especially if the transgression committed was against the ingroup. Future researchers could also investigate whether children perceive that transgressors with ambiguous intentions act more purposefully when aggression is committed toward members of their gender outgroup vs. gender ingroup.

Although the present depictions of relational and physical aggression were based on previous literature, it is unclear if both story types conveyed intentionality information to the same extent. It is possible that physical aggression was more readily perceived as accidental in the ambiguous condition, at least compared to relational aggression (i.e., ignoring someone and walking away from them on purpose vs. with ambiguous intent). Therefore, even though we manipulated intent by the inclusion of “on purpose” (unambiguous condition) or the exclusion of “on purpose” (ambiguous condition), the relational story content could have inadvertently conveyed intent information, above and beyond our intent manipulation.

Most importantly, there are a multitude of other cues that children might also consider, such as race, how frequently the transgressor partakes in aggression, or if the transgressor was acting in retaliation. Future studies should build on the present findings by including these and other relevant cues. It is also critical for future research to include a more diversified sample (e.g., race, ethnicity, socioeconomic status), as beliefs might not be uniform across all groups.

## Conclusion

The present study investigated how children prioritize and make use of different contextual cues—aggression form, transgressor gender, and transgressor intentionality—in aggression scenarios to guide their sociomoral reasoning, along with consideration for how dependence on these cues changes between 5 to 10 years of age. The present research reveals that not all contextual cues were treated equally. Only aggression form and transgressor intentionality were impactful to children’s sociomoral judgments: physical transgressors with unambiguous, purposeful intent were judged more harshly than those with ambiguous intent, yet intentionality did not impact judgments about relational transgressors. Importantly, transgressor gender changed children’s social preferences. This implies that children value different contextual cues to guide their moral judgments, which are reflective of behaviors and actions, compared to their social preferences, which are reflective of their attitudes about each transgressor. The findings from this study likely extend to how children navigate issues in their own friendships and subsequently form moral judgments about their peers: aggression form and transgressor intentionality are valued over transgressor gender.

## Data Availability Statement

The raw data supporting the conclusions of this article will be made available by the authors, without undue reservation.

## Ethics Statement

The studies involving human participants were reviewed and approved by the Office of Research Integrity – Institutional Review Board at the University of North Carolina at Greensboro. Written informed consent to participate in this study was provided by the participants’ legal guardian/next of kin.

## Author Contributions

AY-Y, RC, and JB contributed to the design of the study. AY-Y and RC contributed to data collection. AY-Y, JC, and RC contributed to statistical analyses and wrote sections of the manuscript. AY-Y, JC, and JB contributed to revisions. All authors read and approved the submitted version.

## Conflict of Interest

The authors declare that the research was conducted in the absence of any commercial or financial relationships that could be construed as a potential conflict of interest.

## Publisher’s Note

All claims expressed in this article are solely those of the authors and do not necessarily represent those of their affiliated organizations, or those of the publisher, the editors and the reviewers. Any product that may be evaluated in this article, or claim that may be made by its manufacturer, is not guaranteed or endorsed by the publisher.

## Footnotes

1 Children were also asked to provide justifications for their answers. Because analysis of the justification data supported the presented data and did not add any novel information, we decided not to include it in the final version of the manuscript for the sake of parsimony and clarity.
